# Low Protein Diet Inhibits Uric Acid Synthesis and Attenuates Renal Damage in Streptozotocin-Induced Diabetic Rats

**DOI:** 10.1155/2014/287536

**Published:** 2014-03-13

**Authors:** Jianmin Ran, Jing Ma, Yan Liu, Rongshao Tan, Houqiang Liu, Gancheng Lao

**Affiliations:** ^1^Department of Endocrinology, Guangzhou Red Cross Hospital, Medical College of Jinan University, No. 396 Tong Fu Zhong Road, Guangzhou 510220, China; ^2^Department of Nephrology, Guangzhou Red Cross Hospital, Medical College of Jinan University, No. 396 Tong Fu Zhong Road, Guangzhou 510220, China; ^3^Clinical Institute of Nutrition, Guangzhou Red Cross Hospital, Medical College of Jinan University, No. 396 Tong Fu Zhong Road, Guangzhou 510220, China

## Abstract

*Aim.* Several studies indicated that hyperuricemia may link to the worsening of diabetic nephropathy (DN). Meanwhile, low protein diet (LPD) retards exacerbation of renal damage in chronic kidney disease. We then assessed whether LPD influences uric acid metabolism and benefits the progression of DN in streptozotocin- (STZ-) induced diabetic rats. *Methods.* STZ-induced and control rats were both fed with LPD (5%) and normal protein diet (18%), respectively, for 12 weeks. Vital signs, blood and urinary samples for UA metabolism were taken and analyzed every 3 weeks. Kidneys were removed at the end of the experiment. *Results.* Diabetic rats developed into constantly high levels of serum UA (SUA), creatinine (SCr) and 24 h amounts of urinary albumin excretion (UAE), creatintine (UCr), urea nitrogen (UUN), and uric acid (UUA). LPD significantly decreased SUA, UAE, and blood glucose, yet left SCr, UCr, and UUN unchanged. A stepwise regression showed that high UUA is an independent risk factor for DN. LPD remarkably ameliorated degrees of enlarged glomeruli, proliferated mesangial cells, and hyaline-degenerated tubular epithelial cells in diabetic rats. Expression of TNF-**α** in tubulointerstitium significantly decreased in LPD-fed diabetic rats. *Conclusion.* LPD inhibits endogenous uric acid synthesis and might accordingly attenuate renal damage in STZ-induced diabetic rats.

## 1. Introduction

Diabetic nephropathy (DN) now is the leading cause of end-stage renal disease in either type 1 or type 2 diabetes. For so many years series of therapeutic strategies have been explored including tightly glucose and blood pressure control, renin-angiotensin blockade [1], and lipid lowering [2]. Unfortunately, these treatments only slow the renal progression rather than reverse the progress [3]. More novel modifiable factors should be sought for DN.

Recently, more and more studies demonstrated that hyperuricemia closely associates with DN progression. In a cross-sectional study, Tseng [4] verified an independent correlation between serum uric acid (UA) and urine albumin excretion in Taiwanese type 2 diabetic patients. In the other cohort study, Hovind et al. [5] found that UA level soon after onset of type 1 diabetes independently predicts the risk for development of DN during a median 18. 1-year follow-up. In an animal study [6], tubulointerstitial injury of diabetic (db/db) mice is significantly ameliorated after treatment with allopurinol for 8 weeks. Clinically, allopurinol therapy for 12 months in hyperuricemic patients with chronic kidney disease (CKD) significantly decreases serum uric acid (SUA) levels and preserves their renal function [7]. Also with allopurinol for 4 months, Momeni et al. [8] proved that urine albumin excretion is significantly reduced in type 2 diabetic patients with DN.

Despite its efficacy for lowering SUA, allopurinol probably has obvious side effects such as severe skin allergy. So hypouricemic agents may not be the optimal option for long-term administration in prevention of DN. It is well established that low protein diet (LPD) intervention effectively and safely attenuates renal damage and retards deterioration of renal function in CKD patients [9]. Yet effects of LPD on DN progression are controversial [10]. Meanwhile, there is no evidence that LPD exerts any influences on endogenous UA metabolism and accordingly improves outcomes of DN or CKD. Therefore in the present study, we investigated effects of LPD on* in vivo* UA synthesis and clearance as well as its possible influences on renal pathology in streptozotocin- (STZ-) induced diabetic rats, an established animal model for diabetes and DN.

## 2. Materials and Methods

### 2.1. Animal Preparation

**Figure 1:**
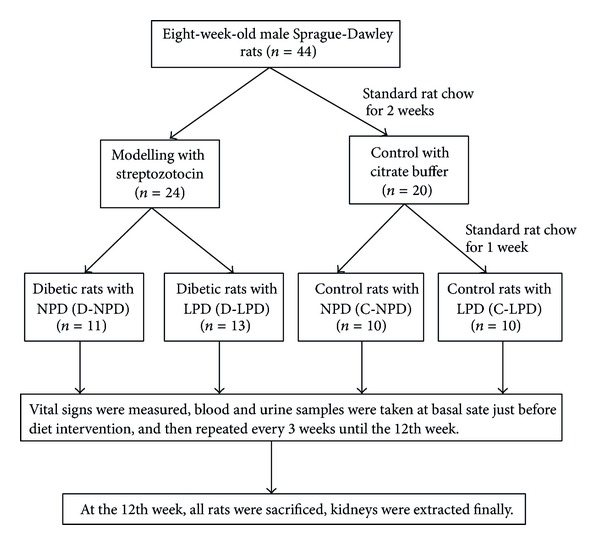
Schematic protocol of the overall animal experiment. NPD: normal protein diet, LPD: low protein diet.

The intact protocol of animal experiment was schematically shown in Figure 1. Specifically, eight-week-old male Sprague-Dawley rats (Guangdong Medical Laboratory Animal Center, China) weighing 180–230 g were adopted. Forty-four rats were collectively housed (2 rats per cage) and fed with standard rat chow for 2 weeks and then randomized into diabetic (*n* = 24) and control (*n* = 20) group. For diabetes formation, rats were intraperitoneally injected STZ (dissolved in 50 mM citrate, PH = 4.2, Sigma, St. Louis, USA) in a single dose of 65 mg/Kg; those with random blood glucose levels over 16.7 mmol/L in three different times were selected for experiments. Control rats were only intraperitoneally injected with the same volume of citrate buffer.

### 2.2. Diet Intervention and Animal Experiments

One week after modelling, rats were further randomized into four diet groups: diabetic rats with normal protein diet (D-NPD, *n* = 11), diabetic rats with LPD (D-LPD, *n* = 13), control rats with normal protein diet (C-NPD, *n* = 10), and control rats with LPD (C-LPD, *n* = 10). Rats in the normal protein diet (NPD) group were fed with foods containing 65% of carbohydrate, 17% of fat, and 18% of protein, while foods in the LPD group are composed of 78% of carbohydrate, 17% of fat, and 5% of protein (Guangdong Medical Laboratory Animal Center, China). Total calorie of per gram of food was the same between NPD and LPD (3.95 Kcal/g). Diet intervention kept on within 12 weeks after modeling, vital signs, blood, and urine samples were collected every 3 weeks till the end of experiment.

Vital signs including systolic blood pressure, diastolic blood pressure, and heart rate were recorded in completely conscious rats by using indirect tail-cuff equipment (LE5002, Harvard Apparatus, USA). After prewarming rats for 20 minutes on a 37°C plate, blood pressure and heart rate of each rat were recorded.

During the whole experiment, all rats had free access to foods and water; room light rotated at a 12-hour light-dark cycle. Twenty-four hours of urine was collected and quantified before the experimental day, while rats were fed in special metabolic cages. On the morning of experiment, foods were withdrawn 12 h before every operation. Rats were sacrificed after 12 w diet treatments; kidneys were removed for histologic and immunohistobiochemical assays. All animal studies were approved by the ethnic committee of Jinan University.

### 2.3. Biochemical Assays

Serum concentrations of glucose, triglyceride, total cholesterol, uric acid (SUA), urea nitrogen (BUN), and creatinine (SCr) were measured by corresponding commercial kits on an automatic biochemical machine (ECHO, ECHO, Italy). Twenty-four-hour urine samples were collected and quantified. Urinary uric acid (UUA), urinary urea nitrogen (UUN) and urinary creatinine (UCr) were detected by the same automatic machine. Urinary albumin was determined by the standard bromocresol green method and 24 h amount of urinary albumin excretion (UAE) was then calculated.

### 2.4. Renal Morphology

Kidneys of all rats were removed and fixed in 4% paraformaldehyde, then embedded in paraffin, and cut into 2 *μ*m sections. The sections were right along dyed by the routine Hematoxylin and Eosin staining method. All slides were digitized and processed by a specific computer system (BX41, Olympus, Japan).

The glomerular area was bordered along the outline of capillary loop and mean glomerular area (MGA) was determined from 15 glomeruli. The extent of mesangial expansion of each group was calculated as sums of the score of each proliferation degree [11]. Tubular damage was evaluated according to the degrees of hyaline degeneration of tubular epithelial cells and quantitatively scored in each group as previously described [12].

### 2.5. Immunohistochemistry

Cellular expressions of tumor necrosis factor *α* (TNF-*α*) and vascular endothelial growth factor (VEGF) in glomeruli and tubules were detected by immunohistochemical assays. Paraffin-embedded sections were deparaffinized and hydrated; endogenous peroxidase activity was completely inhibited by incubation with 3% of perhydrol. A standard two-step immunoperoxidase staining was then performed and negative controls were set by replacing each primary antibody with PBS buffer. The primary antibodies for TNF-*α* and VEGF were both rabbit polyclonal derivations (BOSTER BIO-ENGINEERING, Wuhan, China). Stained glomeruli and tubules were then semiquantitatively scored of tan granule by the computer-assisted light microscopy (BX41, Olympus, Japan) in a scale of 1–4 [13]: no any intracellular tan granule was adjudged as negative and scored as 1, light tan granule in less than 10% of cells was scored as 2, moderate to dark tan granule in more than 60% of cells was scored as 4, and others amid 1–3 were certainly scored as 3. Total score was finally summed from all rats of each group.

### 2.6. Statistical Analysis

Results are expressed as mean ± SD. One-way analysis of variance (ANOVA) and Kruskal-Wallis rank sum test were selected for comparisons of means difference and abnormally distributed data difference, respectively. Global trend difference of parameters measured in a time-course manner was compared by a general linear model for repeated measures. Risk factors for UAE were stepwisely regressed. Statistical difference was accepted as *P* < 0.05.

## 3. Results

### 3.1. General Parameters

As shown in Table 1, as compared with control rats, diabetic rats either fed with NPD or LPD presented with obviously increased daily food intake, water intake and urination. In diabetic rats but not in normal rats, LPD slightly increased daily food intake, water intake, and urine volume (Table 1).

**Figure 2:**
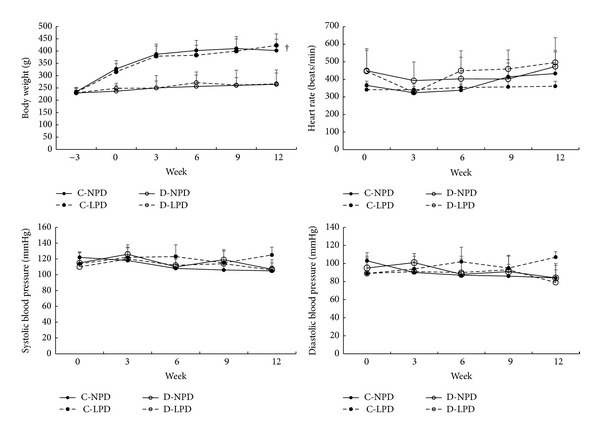
Time course of main vital signs among different rat groups. Heart rate, systolic blood pressure, and diastolic blood pressure were recorded by an indirect tail-cuff method. Solid circle with solid and dashed lines represents C-NPD and C-LPD group, respectively, while hollow circle with solid and dashed lines shows data of D-NPD and D-LPD group, respectively. Data were expressed as mean ± SD. ^†^ *P* < 0.05 for D-LPD versus C-NPD rats in the global trend comparisons.

During the whole experimental course (Figure 2), body weight in either normal or diabetic rats showed increasing trends (*P* < 0.01 for the within group effects in the repeated measure model). Diabetic rats exhibited significant lower body weight than control rats at any time point. Heart rate, systolic and diastolic blood pressure were almost constant during the time course in all rats (*P* > 0.05) and comparable between normal and diabetic rats. NPD and LPD had not any significant effects on these vital signs in either normal or diabetic rats (Figure 2).

### 3.2. Biochemical Characteristics

**Figure 3:**
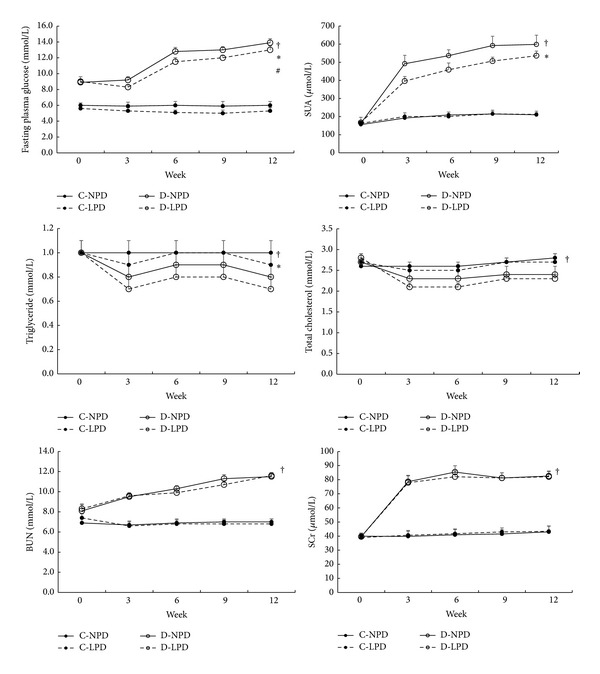
Time course of blood biochemical indexes among different rat groups. Solid circle with solid and dashed lines represents C-NPD and C-LPD group, respectively, while hollow circle with solid and dashed lines shows data of D-NPD and D-LPD group, respectively. Data were expressed as mean ± SD. ^†^ *P* < 0.05 for D-LPD versus C-NPD rats, *P < 0.05 for D-NPD versus D-LPD group, and ^#^ *P* < 0.05 for C-NPD versus C-LPD group in the global trend comparisons. SUA: serum uric acid, BUN: blood urea nitrogen, SCr: serum creatinine.

As shown in Figure 3, fasting plasma glucose was significantly and gradually increased in diabetic rats after modelling (*P* < 0.01). Certainly diabetic rats exhibited remarkably higher plasma glucose than control rats during the whole experiment. Interestingly, LPD slightly lowered fasting plasma glucose in either diabetic or control rats.

Compared with control rats, SUA level of diabetic rats was significantly higher and continuously increased during the experimental period (Figure 3). LPD significantly decreased SUA level in diabetic rats from the 3rd week but exerted no effects in control rats.

Also as shown in Figure 3, STZ-induced diabetic rats showed significantly low levels of triglyceride and total cholesterol from modelling to the end of the study, but LPD further lowered triglyceride instead of total cholesterol level in these rats. No effects of LPD on lipid profiles were found in control rats.

BUN and SCr, two parameters reflecting renal clearance function, were also remarkably increased in diabetic rats after modelling but kept relatively constant from the 6th week. The highest plasma levels of BUN and SCr in diabetic rats were 11.7 mmol/L and 88.1 *μ*mol/L, respectively. LPD did not alleviate both parameters either in diabetic rats or control rats (Figure 3).

### 3.3. Daily Urinary Excretions

**Figure 4:**
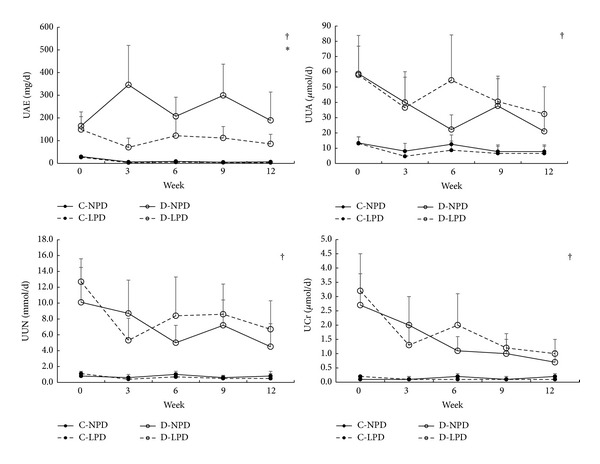
Time course of 24 h urinary excretions among different rat groups. Solid circle with solid and dashed lines represents C-NPD and C-LPD group, respectively, while hollow circle with solid and dashed lines shows data of D-NPD and D-LPD group, respectively. Data were expressed as mean ± SD. ^†^ *P* < 0.05 for D-LPD versus C-NPD rats, *P < 0.05 for D-NPD versus D-LPD group in the global trend comparisons. UAE: urinary albumin excretion, UUA: urinary uric acid, UUN: urinary urea nitrogen, UCr: urinary creatinine.

As shown in Figure 4, daily UAE was significantly increased in diabetic rats after modelling and kept constant during the experimental course. LPD significantly attenuated the high UAE in diabetic rats and had no any effects in control rats.

Daily UUA, UUN, and UCr were also significantly increased in diabetic rats after modelling but tended to be decreased after the 6th week (*P* < 0.01 for within group effects). LPD did not influence these excretions in both diabetic and control rats.

### 3.4. Stepwise Regression for UAE

Among all rats, we defined UAE as the early marker of kidney damage; various risk factors at the 12th week including plasma glucose, systolic, and diastolic blood pressure, total cholesterol, triglyceride, SUA, and UUA were stepwisely regressed. Plasma glucose (*B* = 77.20, 95% CI [6.65, 147.76], *P* = 0.002) and UUA (*B* = 4.40, 95% CI [1.78, 7.02], *P* = 0.033) were finally included in the equation. We so thought that urinary uric acid excretion would be another major risk factor for renal damage in these STZ-induced rats.

### 3.5. Glomerular and Tubulointerstitial Alterations

**Figure 5:**
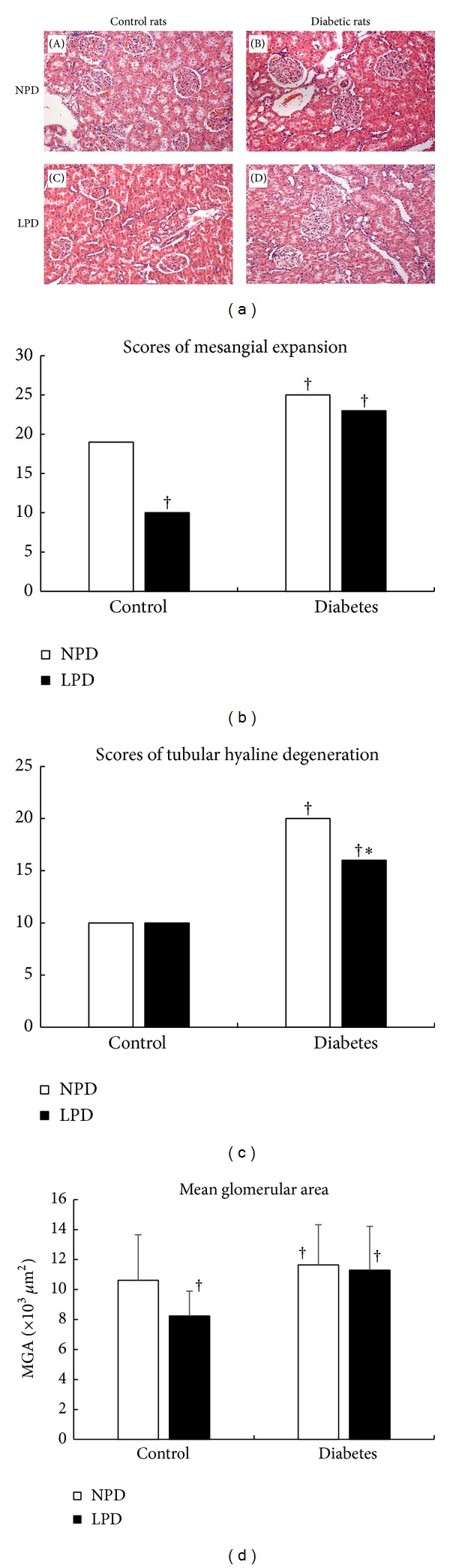
Glomerular and tubulointerstitial alterations in diabetic and control rats. (a) Representative glomerular and tubular structures in the Hematoxylin and Eosin staining. Compared with control rats ((A), (C)), diabetic rats ((B), (D)) showed more severe mesangial expansion and tubular hyaline degeneration. LPD significantly improved mesangial expansion (C) in control rats instead of diabetic rats (D), while it ameliorated the degree of tubular hyaline degeneration in diabetic rats (D) and had no same effects in control rats (C). (b) Quantitative scores of mesangial expansion. (c) Quantitative scores of tubular hyaline degeneration. (d) Alterations of mean glomerular area (MGA) among different groups. LPD reduced MGA in control rats but not in diabetic rats. In (b), (c), and (d), NPD is shown as (□) and LPD is shown as (■). Data in (d) were expressed as mean ± SD. ^†^ *P* < 0.05 compared with C-NPD group. *P < 0.05 was compared with D-NPD group.

As shown in Figures 5(a) and 5(b), diabetic rats either fed with NPD (Figure 5(a)-(B)) or LPD (Figure 5(a)-(D)) exhibited more severe mesangial expansion than control rats (Figures 5(a)-(A) and 5(a)-(C)). Mean glomerular area was also enlarged in diabetic rats (Figure 5(d)). LPD only prevented the mesangial expansion (Figures 5(a)-(C) and 5(b)) and reduced the glomerular size (Figure 5(d)) in normal rats, while it exerted no effects on these alterations in diabetic rats (Figures 5(a)-(D), 5(b), and 5(d)).

As shown in Figures 5(a) and 5(c), STZ-induced diabetic rats either fed with NPD (Figure 5(a)-(B)) or LPD (Figure 5(a)-(D)) also developed into severe tubular hyaline degeneration compared with control rats (Figures 5(a)-(A), 5(a)-(C), and 5(c)). LPD significantly ameliorated the tubular damage in diabetic rats (Figures 5(a)-(D), and 5(c)) but not in these of control rats (Figures 5(a)-(C) and 5(c)).

### 3.6. Expression of Cytokines

**Figure 6:**
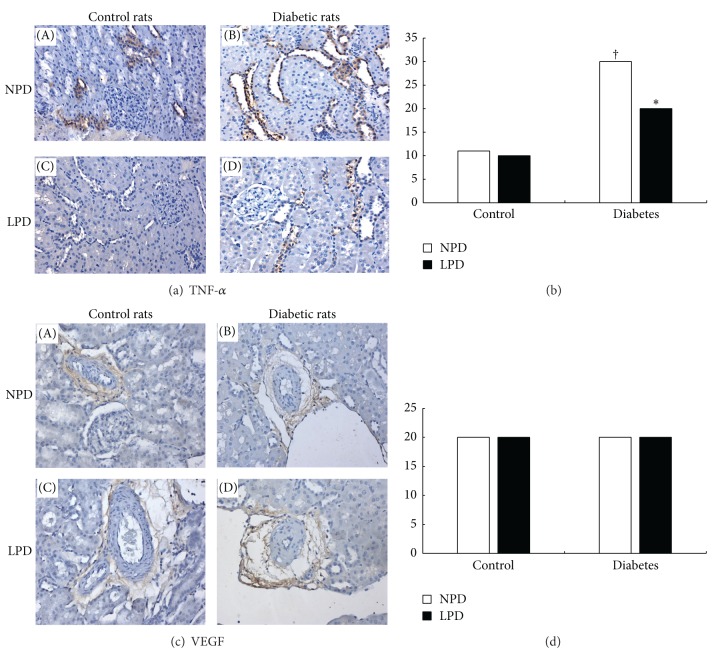
Effects of LPD on tumor necrosis factor *α* (TNF-*α*) and vascular endothelial growth factor (VEGF) expressions in glomeruli and tubulointerstitium. (a) Immunohistochemical staining of TNF-*α*. TNF-*α* mainly expressed in the tubulointerstitial area either in control or diabetic rats. As compared with control rats ((A), (C)), diabetic rats ((B), (D)) expressed more abundant TNF-*α* (the brown color) in this area. LPD only significantly reduced TNF-*α* expression in diabetic rats (d) but not in control rats (C). (b) Quantitative scores of TNF-*α* expression in tubulointerstitium. (c) Immunohistobiochemical staining of VEGF. VEGF occasionally expressed around some arterioles. Diabetic rats ((B), (D)) expressed comparable VEGF as control rats ((A), (C)). LPD exerted no effects on VEGF expressions either in control (C) or diabetic rats (D). (d) Quantitative scores of TNF-*α* expression around arterioles. In (B) and (D), NPD is shown as (□) and LPD is shown as (■).^†^ *P* < 0.05 compared with C-NPD group. *P < 0.05 is compared with D-NPD group.

Two established cytokines for diabetic renal injuries, TNF-*α* and VEGF, were detected by immunohistochemical staining. As shown in Figure 6(a), TNF-*α* expression in tubulointerstitial area was more abundant in diabetic rats (Figures 6(a)-(B), and 6(a)-(D)) than in control rats (Figures 6(a)-(A), and 6(a)-(C)). LPD significantly inhibited TNF-*α* expression in diabetic rats (Figures 6(a)-(D), and 6(b)) but not in control rats (Figures 6(a)-(C), and 6(b)). On the other side, as shown in Figures 6(c) and 6(d), VEGF is casually expressed around some arterioles. There was no difference for VEGF expression between diabetic and control rats. LPD did not alter its expressions in both kinds of rats.

## 4. Discussion

In the present study, STZ-induced diabetic rats developed remarkably hyperuricemia resembling other researches on this model [14, 15]. We further explored the possible underlying mechanisms. Initially, the impairment of renal excretion was assumed because diabetic rats were characterized by increased plasma levels of BUN and SCr, yet daily urinary excretions of uric acid, nitrogen, and creatinine were also significantly increased. Taking all these factors into consideration, we may conclude that hyperuricemia in these diabetic rats is mainly attributed to the increased* in vivo* uric acid synthesis. From our knowledge so far, this is the first study which rendered the fact that uric acid synthesis might be abnormally promoted in diabetes.

Relationship between dietary protein and SUA remains controversial until now [16]. In human, it is well known that red meat intake [17] and sea foods consumption [18] would elevate SUA and increase the incidence of gout, but vegetable protein [19] surely lowers the risk. Despite possible links between different foods and metabolic syndrome, these previous data throw us a light that the quantity and quality of dietary protein may exert different effects on* in vivo* UA metabolism. In a cross-sectional study on healthy young men, Frank et al. [20] proved that constant infusion of the high-protein diet (2.4 g/kg/d) for 7 days significantly increased SUA and daily urinary excretion of UA compared with the normal-protein diet (1.2 g/kg/d). As to the present study, LPD to diabetic rats significantly decreased SUA from the 3rd week to the end of the experiment, while it had no effects on daily UUA, UUN, and UCr overall. Levels of BUN and SCr also were not decreased after LPD intervention. So accordingly, we believe that LPD lowers SUA by inhibiting* in vivo* uric acid synthesis instead of increasing its urinary excretion in STZ-induced diabetic rats.

For its various and affirmative evidences on slowing the progression of renal function loss [21, 22], LPD has been widely recommended as the important nutritional management for CKD [23]. Yet by so far, results of LPD intervention on renal function in DN patients are controversial [24–26]. In this animal study, STZ-induced diabetic rats became glomerular hyperfiltration with early renal function loss according to the following biochemical illustrations: elevated UUN, UCr, and UAE, as well as increased serum BUN and creatinine. Diabetic rats also developed into enlarged glomeruli and expanded mesangium. Simultaneously, obvious tubular injuries marked as significant tubular hyaline degeneration and TNF-*α* infiltration were found. This is not unusual, DN mainly manifests with glomerular damages but associates with tubular injuries [27, 28] such as tubular proliferation and tubulointerstitium macrophage infiltration. Interestingly, LPD decreased UAE level significantly but leave serum BUN and creatinine unchanged, MGA and mesangial expansion in diabetic rats were not affected by LPD intervention too. On the other hand, LPD improved the degree of tubular hyaline degeneration and TNF-*α* expression in tubulointerstitium of diabetic rats. In sum, we might consider that LPD had no effects on glomerular structure and hyperinfiltration state, while it attenuated tubular injury in diabetic rats.

Inflammation plays the pivotal role in diabetic tubular injury [29, 30]. Recently macrophage infiltration in renal interstitium has drawn more and more attention for its causative actions to DN [31]. Intrarenal macrophages are classically attracted by monocyte chemoattractant CC chemokine ligand 2 (CCL2) via its receptors (CCR2) [32], and antagonists of CCL2 and CCR2 have been proved to reduce interstitial macrophage recruitment and ameliorate interstitial fibrosis in diabetic animal models [33, 34]. Whether LPD exerts any effects on this process remains unclear. VEGF is a cytokine which promotes angiogenesis and is therefore involved in the process of diabetic microvascular complications [35]. In our study we did not find any difference about renal VEGF expression between diabetic and normal rats; the main causes might be attributed to short observation period and without any hypoglycemic treatment. Nevertheless, recent population studies in French and Danish [36] on two main genetic variants of VEGF also do not establish any relations between VEGF and T2DM as well as its microvascular complications.

The last hypothesis of the present study is whether LPD attenuated these diabetic renal damages through the improvement of uric acid metabolism in a way. In the stepwise regression, UUA was another independent risk factor for UAE besides fasting plasma glucose. By now, we still do not know how hyperuricemia directly affects the pathogenesis of DN. Several previous researches disclose some possible links between uric acid and renin-angiotensin-aldosterone system and proinflammatory pathways [37]. Among them, the persuasive one is that uric acid, as the crystals from cellular necrosis, can activate inflammasome NLRP3, which consequently trigger caspase-1 and its downstream cytokines including IL-1*β* and IL-18 [38]. IL-1*β* and IL-18 have been proved potentially expressed on tubular epithelial cells and may closely relate to uric acid induced interstitial damage [39]. On the other hand, a latest research [40] in patients with gout and asymptomatic hyperuricemia shows us that hyperuricemia leads to elevated serum CCL2 and monocyte recruitment. All these data illustrate the possibility that uric acid may play an important role in proinflammatory diabetic renal damage, especially in tubular injury.

Similar to other animal studies [6, 13, 41, 42] which solely aimed at inhibiting uric acid synthesis by xanthine oxidase inhibitor, LPD in this study mainly decreased UAE and attenuated tubular injury of STZ-induced rats. To some extent, we may speculate that LPD inhibits* in vivo *uric synthesis so that ameliorates diabetic tubular damages. But. for the complicated causes of DN, the present study still could not precisely answer the question how LPD benefits diabetic renal injuries through affecting uric acid metabolism. More basic and clinical researches are required in this field.

## 5. Summary

STZ-induced diabetic rats present with obviously increased* in vivo* uric acid synthesis and renal damages. LPD intervention significantly inhibits the high level of uric acid formation and attenuates daily UAE as well as tubulointerstitial damages. Whether decreased SUA is partly responsible for improvements of renal function and morphology in LPD treatment remains to be determined.

## Figures and Tables

**Table 1:** Mean daily food intake, water intake and urine volume for one rat during the whole period after dietary intervention.

	*N *	Food intake (g/day)	Water intake (mL/day)	Urine volume (mL/day)
C-NPD	11	15.9 ± 5.0	24 ± 5	9 ± 6
C-LPD	13	20.4 ± 4.1	25 ± 4	9 ± 2
D-NPD	10	29.1 ± 6.7^†^	127 ± 24^†^	93 ± 32^†^
D-LPD	10	35.7 ± 7.4^†∗^	174 ± 30^†∗^	98 ± 38^†∗^

Data are expressed mean ± SD. ^†^ *P* < 0.05 versus C-NPD group, *P < 0.05 versus D-NPD group.
